# Pilot analysis of protein profile alterations in plasma and aortic tissue of spontaneously hypertensive rats

**DOI:** 10.3389/abp.2026.16571

**Published:** 2026-05-21

**Authors:** Anastasios Papageorgiou, Fragkiski-Ioanna Sofiou, Anastassios Philippou, Maria Gkrampovari, Konstantinos Agiannitopoulos, Lubomir Traikov

**Affiliations:** 1 Department of Medical Physics and Biophysics, Medical University - Sofia, Sofia, Bulgaria; 2 Medical School, Department of Physiology, National and Kapodistrian University of Athens, Athens, Greece; 3 Department of Chemistry, National and Kapodistrian University of Athens, Athens, Greece; 4 Division of Genetics and Biotechnology, Department of Biology, National and Kapodistrian University of Athens, Athens, Greece

**Keywords:** blood plasma and aorta tissue, gel electrophoresis, hypertension, proteome analysis, spontaneous hypertensive rats (SHRs)

## Abstract

The role of proteins in cellular activity has received close attention. Proteome analysis can detect early enough changes in the cell before the appearance of pathological changes. Due to the increasing number of cardiovascular complications, more specifically hypertension in modern life, this experimental pilot study tries to detect early changes in the protein profile in cases with arterial hypertension. In this pilot study, animal models were used, specifically control rats and spontaneously hypertensive rats (SHRs), which are widely used as translational models relevant to human cardiovascular physiology. Using gel electrophoresis (GE) method, we analysed proteomic signature of blood plasma and aorta tissue samples of the animal models.

## Introduction

Proteins are the main structural and functional components of cells created by the sequence of single components called amino acids (Ling et al., 2023). Proteome analysis uses the protein constitutions of living cells so as to evaluate very early changes in the living cells before structural changes appear, on the border with pathological and normal fractures (Xie et al., 2025).

According to scientific literature, there are a number of different methods, including immunohistochemistry, enzyme-linked immunosorbent assay, MALDI-TOF/TOF, Western blot analysis, affinity chromatography and Sodium Dodecyl Sulfate-Polyacrylamide Gel Electrophoresis (SDS-PAGE) able to give us data to understand the biomolecular pathways in which proteins participate (Masson and Pashirova, 2025). These techniques are based on purification analysis, analysis of structural components and quantification of several protein molecules.

SDS-PAGE is an effective method for studying protein isoforms, post-translational modifications, and allelic variants, areas that are critical in responding to stress stimuli (Jorrin-Novo et al., 2019). The process involves applying an electric field to a gel matrix, where the molecules are charged and migrated at different rates inversely proportional to their size. PAGE is suitable for protein separation, while agarose gel electrophoresis is preferred for DNA fragment analysis. The method is highly used not only due to lower cost compared to other separation techniques, but also due to simplicity, and broad applicability (Santos, 2023). The technique uses fluorescence to label proteins. Multiple samples can be separated in a single gel, which improves productivity and reduces variability in proteomic studies. It is used in identification of protein expression under different conditions, like treatment responses and diseases (Cramer and Westermeier, 2012).

Protein homeostasis is a very important process for the normal cellular homeostasis (Chen et al., 2023). Their disturbances can significantly change cardiovascular function and adaptation to different stimuli such as high blood pressure (Mainali et al., 2023; Tadic et al., 2020; Hou and Yang, 2024). Hypertension is one of the major problems in the modern way of life. The main problem is increase in blood pressure within the arteries, which contributes to significant arterial damage and consequently increased work for the myocardium (Chironi et al., 2003; Taghizadeh et al., 2015; Taghizadeh et al., 2022; Agafonova et al., 2023). Proteostasis mechanisms are closely related with hypertension, even if the patient is undergoing medical treatment with well controlled blood pressure (Teixeira et al., 2022; Al Ashmar et al., 2024).

SHRs were developed in 1963 by Okamoto through selective breeding for hypertension by mating two rats with pre-existing hypertension. Hypertension is developed as a result of a single gene mutation (Okamoto and Aoki, 1963). At the same time, neurological implications such as cognitive impairment and brain atrophy, are observed, reflecting features of hypertension -associated brain disorders in humans (Tayebati et al., 2012). Recently, an increasing number of animal models are created in order to investigate the exact pathway of many cardiovascular diseases related to hypertension (Yu and Shang, 2013; Breckenridge, 2013). SHRs model is a unique model due to the similarity with human essential hypertension and Wistar rats as a control animals are a useful tool for studying the pathogenesis of the disease and experimental strategies that are impossible to run in humans (Muñoz-Durango et al., 2016; Pezeshki and Nematbakhsh, 2021). Furthermore, several studies have tried to investigate the exact molecular pathophysiology of hypertension as well as the disturbances in renin-angiotensin-aldosterone system which contribute to vascular pathology and increased cardiac morbidity and mortality (Ames et al., 2019; Lip and Padmanabhan, 2020).

In the present pilot animal model study, we used control rats and SHRs trying to investigate the main differences in protein profiles using SDS-PAGE.

## Materials and methods

The current investigation were based on the evaluation of hypertension and how hypertension is possible to alter the band distribution of proteins into blood plasma, heart and aorta. Our study was based on explanation of stress undergone by the myocardium and aorta through evaluation of vascular structure and protein materials from animal models more precisely from rats (n = 2). This pilot study is a descriptive, exploratory investigation using rats, specifically one rat from the control group (Wistar) (n = 1) and one rat from the SHR model group (n = 1) to conduct preliminary experiments examining the proteomic signature of heart tissue, aorta and blood plasma. The rat models were male, around 350 g and 3 months old.

A descriptive and highly accurate and similar protocol was done in both rats following the exact same procedure to the animals able to isolate blood primarily and then to proceed to extraction of heart and aorta. The animals were anesthetized using 15 mg/kg xylazine and 85 mg/kg ketamine for achieve extended analgesia and immobility able to proceed to blood collection (Van Pelt, 1977; Wixson et al., 1987). The process of isolation of blood plasma required the collection of the blood and the centrifugation of it at 2,500 × g for 15 min at room temperature (20 °C). After that, the supernatant yellow plasma was aspirated from the rest of the sediment (blood cells) and it was stored in Eppendorf tubes in −20 °C. Then, we opened the ventral portion of the animal using a scalpel, to isolate the heart and extract the aorta. In the process of isolating the aorta, aorta was removed as a whole vessel, from directly above the heart (ascending aorta) to its bifurcation into the common iliac arteries (aortic arch, thoracic and abdominal aorta) and we noticed a thick and rigid aortic wall and heart of SHR compared with the control. Then the organs were placed in physiological solution for washing. Next step was the clearance of the aorta and separation of the middle layer of tunica media from the external layer of tunica adventitia. Then the tunica media was diluted in phosphate buffer solution (PBS) with pH 7.4 following Cold Spring Harbor protocols (Laemmli, 1970) and it was centrifuged in 2,500 × g in order the proteins to be released into supernatant. The same protocol of dilution was followed also in blood plasma samples. Then protein analysis was subsequently performed with gel electrophoresis so as to evaluate proteins into blood plasma and aorta and more precisely to estimate an approximate concentration of proteins per group and any possible differences between them through diagrammatic presentation. Previous experience and experiments with these types of rats have proven that the experimental accuracy is based not a high number of animals but to repetition of the same experiment twice under scientifically controlled conditions. Based on that we proceed to the electrophoresis experiment twice proving the same result in gel formation and diagrammatic appearance.

After visualizing the gel by staining with Coomassie Brilliant Blue, we dried it to remove the remaining liquid allowing it to be scanned using a gel scanner for analysis by digital densitometry. Densitometry evaluated protein levels in the obtained gels and the results were subsequently analyzed using ORIGIN Pro software for data processing and interpretation. To achieve a more objective analysis of the obtained gel densitometry was performed of the gel images. Densitometric data are expressed in arbitrary units (a.u.), reflecting relative band intensity, and were calibrated using a BSA standard curve for approximate protein quantification.

As the current analysis represents a preliminary pilot investigation with a very limited sample size (n = 2), the analysis was restricted to descriptive observations. No inferential statistical tests were conducted, as the small number of animals does not provide sufficient power to support meaningful statistical comparisons. Finally, the present study is recognized and has the permission from the ethic committee of Medical University of Sofia (MUS) related to animal model use following the rules about animal development and use.

## Results

In the course of our study, we monitored changes in the protein distribution profiles isolated from the endothelial and smooth muscle layer of the aorta in control and SHR model. All densitometric profiles are presented with molecular weight (kDa) on the x-axis and densitometric intensity (arbitrary units, a.u.) on the y-axis, representing relative protein abundance.

**FIGURE 1:**
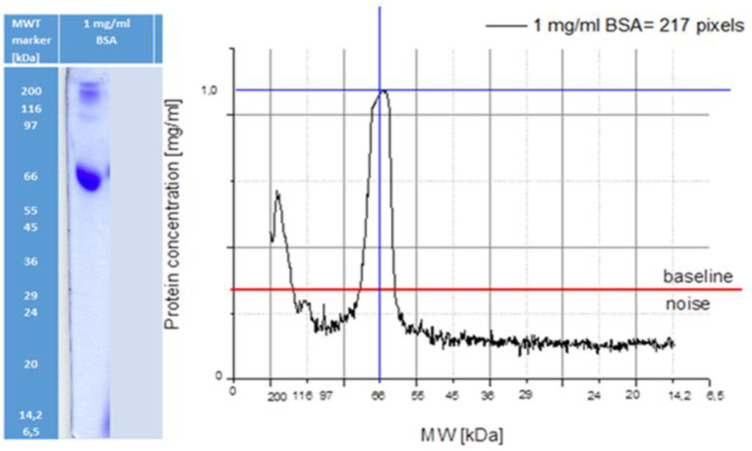
SDS–PAGE densitogram of Bovine Serum Albumin (BSA) standard (1 mg/mL). The x-axis represents protein molecular weight (kDa), while the y-axis represents densitometric intensity (arbitrary units, a.u.), corresponding to protein concentration. A reference value of 217 pixels corresponds to 1 mg/mL protein concentration at 66 kDa, and was used for calibration of protein quantification.

Following SDS-PAGE, we visualized proteins to determine their length and abundance. After that we performed a standardization method including PAGE-densitometry of standard solution of 1 mg/mL BSA. Densitometry estimated not only molecular weight which is determined preliminary by the position of protein markers but also, is able to read the amount of protein in the medium for calculating the integral of the curve determined after densitometry of the obtained individual bands. In Figure 1, it is shown that a calibration curve was established using BSA, where 217 densitometric units (pixels) correspond to a protein concentration of 1 mg/mL at 66 kDa, allowing semi-quantitative estimation of protein abundance.

**FIGURE 2:**
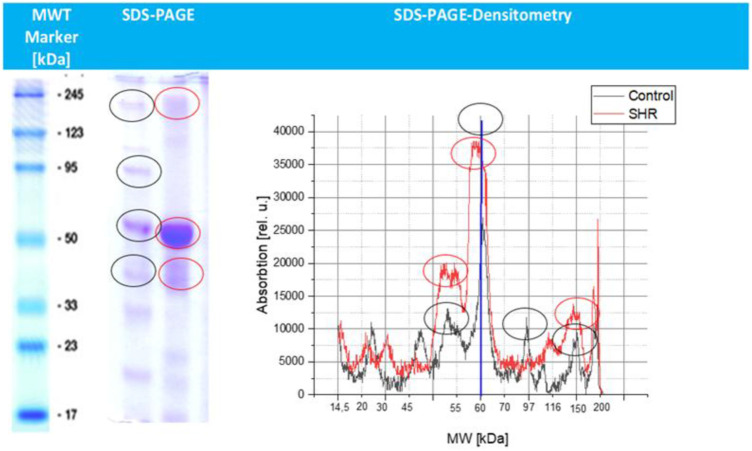
SDS–PAGE densitometric profile of blood plasma proteins from control and SHR animals. The x-axis shows protein molecular weight (kDa), and the y-axis shows densitometric intensity (arbitrary units, a.u.), reflecting relative protein abundance. The blue line represents the control rat, while the red line represents the SHR. Major protein bands and their approximate molecular weights are indicated in the profile.

Additionally, in Figure 2 is described the result of the proteomic pattern observed in the control rat (black line) and SHR (red line) after isolation of blood plasma supernatant taken from both animals. Profile of distribution of control rat is much more stable and it is dominated by one protein band, at 60 kDa and four less pronounced bands at 25 kDa, 50 kDa, 95 kDa and 150 kDa respectively. On the other hand, SHR proteomic pattern is appeared with higher concentration in comparison to control and a more dispersed protein distribution dominated by one protein band at 60 kDa and three less pronounced bands at 25 kDa (similar to control), 50 kDa appeared as double band distribution and with definitely higher concentration and at 150 kDa respectively, after recalculation of concentration equal to a concentration of 0.83 mg/dL.

**FIGURE 3:**
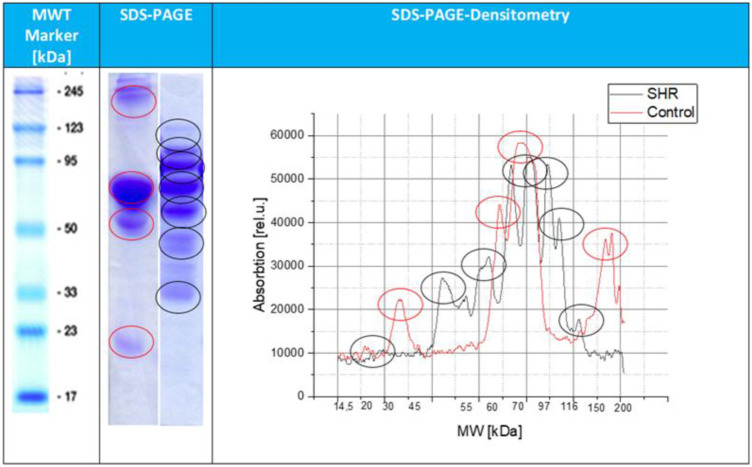
SDS–PAGE densitometric profile of aortic tissue (tunica media) proteins from control and SHR animals. The x-axis represents protein molecular weight (kDa), and the y-axis represents densitometric intensity (arbitrary units, a.u.), corresponding to relative protein levels. The red line corresponds to the control rat, while the black line corresponds to the SHR. Prominent protein bands are annotated according to their molecular weight.

At Figure 3 is shown the result of the protein distribution profile in SHR, which is dominated by 7 protein bands. The dominated four protein bands, located at 96 kDa band (4 mg/dL), a second band of 64 kDa (8 mg/dL) a third band of 58 kDa (5 mg/dL) and a fourth band at 48 kDa (5 mg/dL). Moreover, the same graph shows three less pronounced bands at 118, 110 and 22 kDa respectively (after recalculation equal to concentrations of approximately 0.93 mg/dL). On the other hand, the control protein distribution profile is dominated by a single band at 66 kDa and four less pronounced bands at 180 kDa, 60 kDa, 40 kDa and 22 kDa. The presence of multiple protein bands in comparison to the relatively stable profile of the control rat, reflects increased cellular activity, possible vascular injury and susceptibility to bleeding and tissue adaptation processes.

Lastly, based on tissue structure and composition SHR’s aorta was shorter and much more rigid relative to the control. Importantly, there was a higher stiffness into heart and vessel resulting in thicker vascular wall and thicker myocardium.

## Discussion

In the present pilot animal model study, we observed two important consequences of arterial hypertension based on macroscopic and microscopic evaluation. On the one hand, arterial hypertension affects significantly the structure of aorta causing changes to its elasticity leading to vascular stiffness and remodelling. On the other hand, arterial hypertension is associated with protein profile instability and consequently due to genetic profile instability. This proteomic imbalance leads to different expression of proteins between control rat and SHR proven by our electrophoresis results. The interesting investigation is that proteomic disturbances occur both to animals’ blood plasma in a smaller degree and to animals’ aortic tissue, especially in muscular layer of aorta, which we detect the most severe and significant differences. As it is observed, SHR animals try to adapt to the mechanical stressful stimuli and the whole organism tries to reshape structure and properties of vital organs such as heart and aorta. However, these properties lead to anatomical changes such as heart enlargement and aortic stiffness in order to maintain cardiac output as well as aortic pulse wave able to push the blood to the periphery. These changes involving alteration of mechanical properties of cardiovascular system were confirmed by molecular analysis of proteins. More precisely, relative abundance differences in SHR rat detected by SDS-PAGE analysis, suggesting early molecular changes that can be involved in complex vascular remodelling and dysfunction that arise in chronic hypertension situations leading additionally to chronic inflammatory progression with secretion of inflammatory factors such as C-reactive protein, cytokines and chemokines as well as several immune and inflammatory cells causing endothelial dysfunction as well as cellular and muscular disorganization (Xiao and Harrison, 2020). Although subsequent proteomic pattern changes are understood in the attempt of organism to respond to vascular stress, the protein distribution in blood and aortic tissue respectively is highly irregular as shown in diagrams. It is obvious that these changes are followed by an altered genetic background thereby causing expression of different proteins at varying concentrations spontaneously. However, despite their adaptation to these alterations, SHRs generally, have a substantially shorter lifespan. Even hypertension in SHR model has genetic origin and the rats are familiar to such vascular forces; they live for shorter period of time (Ames et al., 2019).

It is widely acknowledged that hypertension exerts sustained mechanical stress on vascular endothelial and smooth muscle cells, particularly in the tunica media which is the middle layer of the vessel created a complex network of smooth muscle cells able to adapt and withstand blood pressure forces (Lip and Padmanabhan, 2020). This biomechanical stress triggers several intracellular signaling pathways that cause changes in the expression of genes and proteins as well as the production of reactive oxygen species (ROS) (Touyz and Schiffrin, 2004). The observed increase that was discovered in band intensity in the 50–60 kDa range in SHR plasma likely corresponds to elevated levels of albumin and other stress-responsive proteins such as haptoglobin and transferrin, which have been linked to inflammation and oxidative stress in hypertensive conditions (Virdis and Taddei, 2011; Fujita and Fujita, 2013).

In the aortic tissue sample belonging to SHR, the protein distribution shown new dominant bands in the range of 58–96 kDa, suggesting upregulation of proteins and proteome profile at all (Intengan and Schiffrin, 2001; Montezano and Touyz, 2012). All those investigations have shown changes between SHR animal and control ones, but it is necessary to provided additional sophisticated investigations able to give us more data. The value of the present study is that using a small number of animals, we achieved to find and to detect significant changes was done in protein profile of SHRs in comparison to normotensive rats. In addition, there is better understanding of the effects of hypertension to humans while these preliminary data can give as new perspectives for more experiments and blood tests and understanding in humans suffered by arterial hypertension through analysis of their protein profile from blood. A future bridging study from the present animal study to a human may lead to development of better and more targeted antihypertensive therapies based on proteomic and subsequently the genetic profile of each patient decreasing all the adverse effects followed by the increase of arterial blood pressure.

Previous studies have shown that hypertensive stimuli not only affect the mechanical properties of vessels but also induce metabolic shifts and chronic low-grade inflammation, characterized by the expression of inflammatory factors such as several cytokines and lymphocytes (Schiffrin, 2014). The altered protein expression pattern we found in our study may represent these early pathophysiological processes. For instance, the increased presence of low-molecular-weight proteins (e.g., at ∼48 kDa) could indicate activation of inflammatory mediators or degradation fragments from larger structural proteins, possibly due to enhanced proteolytic activity in the hypertensive vascular wall (Zhou et al., 2012).

Finally, it is broadly accepted that high blood pressure negatively affects a number of organs and systems in the body. Hypertension is closely related to coronary artery disease, renal complications, neurovascular disorders as well as hemodynamic imbalances (Rosendorff, 2007; Pistoia et al., 2016). These effects lead to a dramatic increase in cost for healthcare system due to increased morbidity and mortality. The results of the present study support the use of proteomic approaches in identifying early molecular biomarkers of hypertension, providing valuable insights into post-translational modifications, protein-protein interactions, and the downstream effects of biomechanical stress on vascular homeostasis (Rappsilber et al., 2007; Aslam et al., 2017).

While this pilot study successfully identified distinct alterations in the protein profiles of both plasma and aortic tissue in SHR, it is important to acknowledge its limitations. The primary limitation is inherent to the SDS-PAGE methodology used; although it is highly effective for separating proteins by molecular weight and revealing quantitative changes, it does not allow for the definitive identification of the specific proteins that constitute the differential bands observed. Furthermore, this investigation focused on proteomic profiling and lacked functional or clinical correlates, such as direct measurements of vascular reactivity, inflammatory cytokine levels, or gene expression data. Incorporating such analyses would provide a more comprehensive pathophysiological context for the protein changes.

To directly address these limitations and build upon these promising initial findings, we have outlined a clear trajectory for future research. Our immediate next step is to employ high-resolution tandem mass spectrometry (LC-MS/MS) to unambiguously identify the candidate proteins responsible for the significant bands observed in the SHR samples. Following identification, we will develop targeted assays, such as Western blot analysis or enzyme-linked immunosorbent assays (ELISA), to validate the expression levels of these specific proteins in a larger, independent cohort of animals. This subsequent phase will also integrate detailed physiological and clinical parameters, including hemodynamic measurements, histological assessment of vascular remodeling, and analysis of oxidative stress markers. We anticipate that this integrated approach will not only validate the potential biomarkers discovered here but also elucidate their functional roles in the development and progression of hypertension, ultimately contributing to the identification of novel therapeutic targets.

In conclusion, this experimental pilot study reinforces the potential of proteomic analysis to detect early molecular changes in hypertension. The observed alterations in protein expression patterns in SHR reflect both systemic (plasma) and local (aortic) responses to elevated blood pressure. These findings support the need for further proteomic analysis with LC-MS/MS and functional studies to better characterize the pathophysiological mechanisms of hypertension and cardiovascular disease.

## Data Availability

The datasets presented in this study can be found in online repositories. The names of the repository/repositories and accession number(s) can be found in the article/supplementary material.
